# 3D Domain Adaptive Instance Segmentation via Cyclic Segmentation GANs

**DOI:** 10.1109/JBHI.2023.3281332

**Published:** 2023-08-07

**Authors:** Leander Lauenburg, Zudi Lin, Ruihan Zhang, Márcia dos Santos, Siyu Huang, Ignacio Arganda-Carreras, Edward S. Boyden, Hanspeter Pfister, Donglai Wei

**Affiliations:** John A. Paulson School of Engineering and Applied Sciences, Harvard University, Allston, MA 02134 USA; Department of Informatics, Technical University of Munich, 80333 Munich, Germany; John A. Paulson School of Engineering and Applied Sciences, Harvard University, Allston, MA 02134 USA; Media Lab, MIT, Cambridge, MA 02139 USA, E. S. Boyden is also with the HHMI, Chevy Chase, MD 20815 USA; Computer Engineering Program, University of the Rio dos Sinos Valley, São Leopoldo, RS 93022-750, Brazil. Work was done at Harvard University; John A. Paulson School of Engineering and Applied Sciences, Harvard University, Allston, MA 02134 USA; Department of Computer Science and Artificial Intelligence, University of the Basque Country ((UPV/EHU)), San Sebastian, Spain, Ikerbasque, Basque Foundation for Science, Bilbao, Spain and Donostia International Physics Center (DIPC), San Sebastian, Spain; Media Lab, MIT, Cambridge, MA 02139 USA, E. S. Boyden is also with the HHMI, Chevy Chase, MD 20815 USA; John A. Paulson School of Engineering and Applied Sciences, Harvard University, Allston, MA 02134 USA; Computer Science Department, Boston College, Chestnut Hill, MA 02467 USA

**Keywords:** 3D Instance Segmentation, Unsupervised Domain Adaptation, Expansion Microscopy (ExM), Electron Microscopy (EM), Zebrafish, Neuronal Nuclei

## Abstract

3D instance segmentation for unlabeled imaging modalities is a challenging but essential task as collecting expert annotation can be expensive and time-consuming. Existing works segment a new modality by either deploying pre-trained models optimized on diverse training data or sequentially conducting image translation and segmentation with two relatively independent networks. In this work, we propose a novel *Cyclic Segmentation* Generative Adversarial Network (CySGAN) that conducts image translation and instance segmentation simultaneously using a unified network with weight sharing. Since the image translation layer can be removed at inference time, our proposed model does not introduce additional computational cost upon a standard segmentation model. For optimizing CySGAN, besides the Cycle-GAN losses for image translation and supervised losses for the annotated source domain, we also utilize self-supervised and segmentation-based adversarial objectives to enhance the model performance by leveraging unlabeled target domain images. We benchmark our approach on the task of 3D *neuronal nuclei* segmentation with annotated electron microscopy (EM) images and unlabeled expansion microscopy (ExM) data. The proposed CySGAN outperforms pre-trained generalist models, feature-level domain adaptation models, and the baselines that conduct image translation and segmentation sequentially. Our implementation and the newly collected, densely annotated ExM zebrafish brain nuclei dataset, named *NucExM*, are publicly available at https://connectomics-bazaar.github.io/proj/CySGAN/index.html.

## I. Introduction

**Fig. 1.**
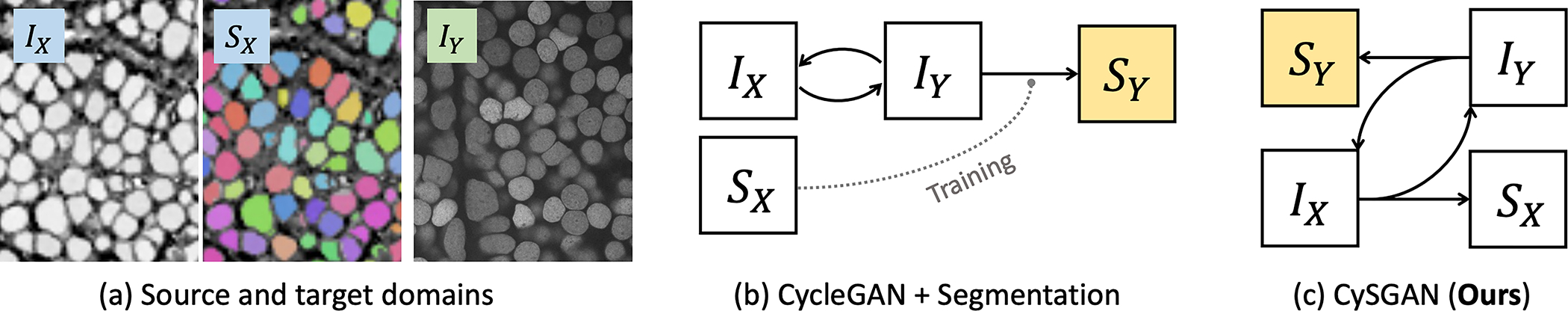
Overview of the task and methods. (**a**) We aim to segment 3D instances in a completely unlabeled target domain $\left(I_{Y}\right)$ by leveraging the images $\left(I_{X}\right)$ and masks $\left(S_{X}\right)$ in the source domain (*i.e., unsupervised* domain adaptation). Instead of (**b**) conducting image translation (*e.g*., via CycleGAN [9]) and instance segmentation as two separate steps, we propose (**c**) Cyclic Segmentation GAN (CySGAN) to unify the two functionalities using weight sharing, which is optimized with both image translation as well as supervised and *semi-supervised* segmentation losses.

The 3D Instance segmentation of cell nuclei is an essential topic attracting both biomedical and computer vision researchers [1]–[5]. Supervised deep learning with in-domain annotations (*e.g*., U-Net [6], [7]) has become the dominant methodology for mainstream imaging modalities. However, such an approach is less applicable for novel imaging modalities, *e.g*., expansion microscopy (ExM) [8]^1^, due to the lack of existing labels and the high annotation costs for newly collected data. This work focuses on segmenting a new imaging modality without any in-domain annotation (Fig. 1a).

Two common approaches try to overcome the challenges by leveraging existing labels from mainstream domains. One approach is to train a supervised model on diverse datasets (*i.e*., a *generalist* model) and apply it directly to the new domain [3], [4]. However, when the domain gap becomes too large, generalist models can produce unsatisfactory predictions without in-domain finetuning that requires new training labels. The other approach, known as unsupervised domain adaptation, usually involves unpaired image-to-image translation models like CycleGAN [9] and segments a new domain with a two-stage pipeline. The first stage translates the source images to match the target domain distribution, aiming to be indistinguishable from the target images while keeping the source structures. The second stage pairs the translated images and corresponding ground-truth labels in the source domain to train a supervised model. The optimized model can then segment real images in the target domain^2^ (Fig. 1b). The limitation of this sequential pipeline is that the segmentation depends on a translation model optimized regardless of the end task. Although recent works improve it by jointly training the translation and segmentation models [10]–[13], the two relatively independent networks still make the pipeline complex.

In this work, we propose a *Cyclic Segmentation* Generative Adversarial Network (CySGAN) that unifies image translation and segmentation to tackle nuclei instance segmentation in an completely unlabeled modality (Fig. 1c). For both the source and target domains, we train a single 3D U-Net [7] that takes only images as input but outputs both segmentation and translated images simultaneously^3^. The segmentation and translation components thus share most of the network weights except for a single output layer. Such a design has two main advantages. First, it decreases the pipeline complexity as we can simply extend a segmentation model with a single additional output channel for image translation to realize domain-adaptive segmentation. Second, the shared backbone implicitly increases the consistency between translated images and predicted segmentation as they share the same input features before the task specific layer. To our knowledge, similar frameworks have been explored only for 2D semantic segmentation (*e.g*., SUSAN [14]) but not 3D instance segmentation that assigns each object a unique index. Furthermore, SUSAN [14] is trained with image translation and supervised segmentation losses. Our CySGAN is additionally optimized with structural consistency and segmentation-based adversarial losses to better leverage the unlabeled domain images, connecting ideas from *semi-supervised* image segmentation.

Moreover, we propose a novel cycle-consistency strategy with data augmentations to improve the performance and robustness of CySGAN. Previous works show that training transformations like blurry, noisy, and missing regions can significantly improve 3D instance segmentation models [5], [15]. However, the image discriminator can easily distinguish between synthesized and real images if the augmentations remain in the translated ones, breaking the balance in GAN training. To tackle this, we proposed to enforce the cycle consistency [9] between the reconstructed images and the clean images instead of the augmented ones, enabling the model to restore corrupted regions during the translation process. This strategy acts as a regularization to improve the spatial awareness of the 3D model as it learns to restore and segment augmented regions using the surrounding context.

To benchmark CySGAN, we curated and annotated two expansion microscopy (ExM) image volumes from a zebrafish brain tissue with dense neuronal nuclei ($I_{Y}$ in Fig. 1a). This dataset is called *NucExM*, with a total of 18.4K instances. These two volumes are complemented by a publicly available and labeled electron microscopy (EM) dataset $\left(I_{X}\right.$ and $S_{X}$ in Fig. 1a). Without any annotation for the ExM domain, our CySGAN outperforms generalist models pretrained on diverse datasets, feature-level adaptation models, and the methods that conduct translation and segmentation using two separate networks. We publicly released our code and the new *NucExM* dataset at https://connectomics-bazaar.github.io/proj/CySGAN/index.html.

### Contributions

We present CySGAN, a novel 3D domain adaptive instance segmentation method that segments instances in an unlabeled domain using a multi-task network. We introduce an augmentation-restoration cycle-consistency strategy that significantly enhances CySGAN’s spatial awareness and robustness without disrupting the generator-discriminator balance. Furthermore, we contribute a new densely annotated ExM zebrafish brain nuclei dataset, *NucExM*, as well as the training and inference code, to the research community.

## II. Related Works

### A. Unpaired Image-to-Image Translation

In biomedical domains, paired images from different imaging modalities are usually expensive or even infeasible to obtain. Therefore, *unpaired* image-to-image translation [9], [16] based on Generative Adversarial Networks (GAN) [17] becomes a sensible methodology to transfer source images to the target distribution. An exemplary framework usually consists of a generator that maps the source images to the target domain and a discriminator that decides whether an input image is from the real target distribution or synthesized. The generator is optimized with the gradients of the GAN loss back-propagated through the discriminator. CycleGAN [9] achieves impressive performance by ensuring *cycle consistency* when transferring translated images back to the source domain using a pair of symmetric generators. Further improvements include shared high-level layers [18] and latent space alignment [10]. We refer readers to the survey by Pang *et al*. [19] for a more detailed discussion of image-to-image translation literature. Specifically, our work combines image translation with segmentation models to tackle unlabeled modalities, extending a standard 3D segmentation with one additional output channel optimized with image translation objectives to adapt to the target distributions. Our proposed CySGAN simplifies existing frameworks that conduct image translation and segmentation using two separate networks.

### B. Instance Segmentation of 3D Microscopy

3D instance segmentation from microscopy images is challenging due to the dense distribution of objects and unavoidable physical limitations in imaging (*e.g*., data is frequently anisotropic with uneven resolution among different axes). Recent learning-based approaches tackle these challenges by first optimizing CNN-based models to predict representations calculated from the instance masks, including object boundary [6], [20], [21], affinity map [15], [22], star-convex distance [4], flow-field [3] and the combination of multiple representations [5]. Watershed transform [23], [24] and graph partition [25] can then be applied to convert the predicted representations into instance masks. However, most existing works train the segmentation models in a supervised learning manner using in-domain annotations, which becomes infeasible considering the cost of acquiring expert annotations for new modalities. Our work focuses on unifying segmentation approaches with image translation to segment instances in new domains via *unsupervised domain adaptation*. At inference time, the image-translation component of CySGAN can be removed, which means CySGAN does not increase the deployment cost upon a standard 3D segmentation model.

### C. Domain Adaptive Segmentation

We focus on *unsupervised* domain adaptation with unlabeled target data. Existing approaches can be categorized into appearance-level and feature-level adaptation methods.

For appearance-level adaptation, utilizing unsupervised image translation is a practical methodology. Chartsias *et al*. [26] designed a two-stage framework that first translates source images to the unlabeled domain using CycleGAN [9] and then trains a separate segmentation model using the synthesized images and source labels. However, since the two modules are optimized independently, the limited awareness of the translation network to the downstream segmentation task can restrict the performance. CyCADA [10], SIFA [13], EssNet [11] and SECGAN [12] improve the sequential model by jointly optimizing the translation and segmentation networks. However, using two separate networks increases the system complexity in training and deployment. The authors of CyCADA [10], for example, stated that although the model is theoretically end-to-end trainable, they need to train it in stages as it is too memory-intensive to optimize the full objective. Different from the mentioned works, we unify image translation and segmentation into a single model to significantly reduce the system complexity. Since the translation and segmentation layers base their predictions on the same high-level features, the CySGAN model enforces the consistency between translated images and segmentation maps from an architectural perspective.

Feature-level adaptation methods commonly optimize a model for two (or more) domains so that the outputs and high-level features from different domains are indistinguishable in distribution. For the unlabeled domain, adversarial losses are usually applied to enforce the alignment. For example, SIFA [13] uses GAN losses to minimize the gap between the segmentation predictions from the real and synthesized target-domain images. Tsai *et al*. [27] designs a model directly taking the source and target images as inputs and applying adversarial losses to align the high-level feature maps. Following existing works, we implement a feature-level adaptation model for 3D instance segmentation and show that our CySGAN and appearance-level adaptation models can achieve significantly better performance in neuronal nuclei segmentation.

To our best knowledge, the only existing work that explores joint translation and segmentation with weight sharing is SUSAN [14], but our work differs from it in two main aspects. First, SUSAN and most works mentioned above are for 2D semantic segmentation, while our work focuses on the more challenging 3D instance segmentation. Second, SUSAN only applies supervised segmentation losses to the annotated domain, while our CySGAN leverages semi-supervised losses for the unlabeled domain in the absence of ground-truth labels.

## III. Method

In this section, we first give an overview of the CySGAN framework (Sec. III-A). We then present the image translation (Sec. III-B) and segmentation (Sec. III-C) objectives to optimize the system, as well as our implementation (Sec. III-D).

### A. The CySGAN Framework

Suppose we have an annotated *source* domain $X=\left(I_{X},S_{X}\right)$ where $I_{X}$ and $S_{X}$ denote the images and paired segmentation labels, respectively. For an unlabeled *target* domain Y with only images $I_{Y}$, the goal is to generate the instance segmentation $S_{Y}$ without acquiring any manual annotations in Y. One straightforward approach is to use some domain adaptation method F to synthesize images $I_{Y^{\prime }}=F\left(I_{X}\right)$ that are *indistinguishable* from the distribution of $I_{Y}$ but keep the instance structure in $S_{X}$. Then a supervised model can be optimized using $\left(I_{Y^{\prime }},S_{X}\right)$ pairs, which predicts $S_{Y}$ from $I_{Y}$ at inference time (Fig. 1b).

*Sequentially* conducting the translation and segmentation suffers from multiple weaknesses. First, the translation model is not designed with an end task in mind and can propagate errors to the second step. Second, the translation model does not benefit from the powerful structural guidance that instance segmentation can impose upon it. Third, two separate modules make the system complicated in training and deployment. Thus, we propose a framework that shares weights between the translation and instance segmentation. Our framework uses two generators - one per domain - that output both translated images and segmentation *simultaneously* (Fig. 1c):

(1) $$F:I_{X}\to \left(I_{Y},S_{X}\right)\;G:I_{Y}\to \left(I_{X},S_{Y}\right)$$

We denote the proposed framework as the *cyclic segmentation* GAN (CySGAN). Specifically, for an image $x_{i}∼I_{X}$, we have $\left[{\overset{ˆ}{y}}_{i},{\overset{ˆ}{x}}_{s}\right]=F\left(x_{i}\right)$, where ${\overset{ˆ}{y}}_{i}$ is the synthesized image, ${\overset{ˆ}{x}}_{s}$ contains the predicted instance representations, and $\left[{\overset{ˆ}{y}}_{i},{\overset{ˆ}{x}}_{s}\right]$ is their concatenation along the channel dimension. For the clarity in the following formulations, we also denote ${\overset{ˆ}{y}}_{i}=F{\left(x_{i}\right)}_{\left[I\right]}$ and ${\overset{ˆ}{x}}_{s}=F{\left(x_{i}\right)}_{\left[S\right]}$. Note that $G\left(F\left(x_{i}\right)\right)$ is no longer a valid expression as both models take only an image as input but output the translated image and segmentation.

**Fig. 2.**
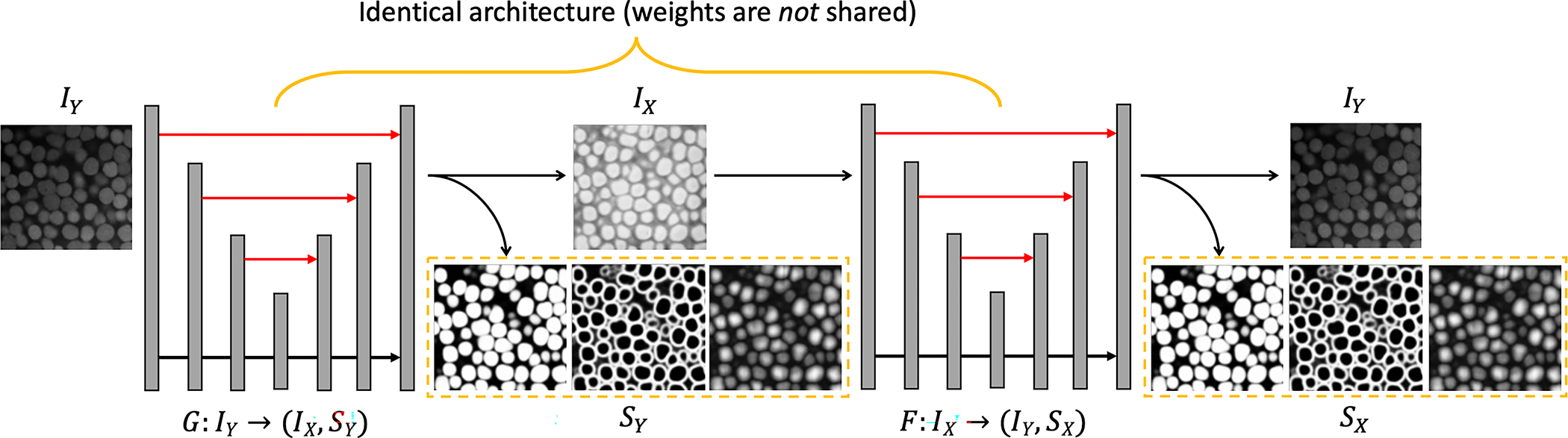
Architecture details of CySGAN. Given an image sampled from $I_{Y}$, the generator G predicts both the transferred image in $I_{X}$ and the BCD segmentation representations $S_{Y}$. Then the generator F takes only the translated image as input and predicts both the reconstructed image and segmentation representations. Specifically, BCD stands for “binary foreground mask, “contour map,” and “distance transform map.” We visualize the predicted BCD representations in the dashed yellow boxes. The two generators have exactly the same architecture, but the weights are *not* shared as they are optimized to translate images in different domains. Only the generator G is needed to segment $I_{Y}$ images at inference time (the output channel for translation can also be removed).

Fig. 2 shows the architecture of our CySGAN framework. For the segmentation part, each of the two generators yields the three instance representations *binary foreground mask* (B), *instance contour map* (C), and *signed distance transform* (D) from which we derive the instance masks (detailed in Sec.III-C). Therefore, a single generator simultaneously outputs the synthesized image and the three instance representations as four different output channels. In particular, ${\overset{ˆ}{y}}_{i}=F{\left(x_{i}\right)}_{\text{[I]}}$ has a single channel while ${\overset{ˆ}{x}}_{s}=F{\left(x_{i}\right)}_{\left[S\right]}$ has three channels, but with the same spatial dimensions (the same for G). Unlike previous works that sequentially conduct image translation and segmentation, our design decreases the system complexity. Moreover, since the translation and segmentation modules base their predictions on the same high-level features in the generator networks, our model implicitly increases the structural consistency between synthesized images and predicted segmentation maps from an architectural perspective.

At inference time, only the generator G is required to segment $I_{Y}$. Besides, the output layer for image translation can be simply removed without influencing the prediction of the segmentation maps. Therefore, our CySGAN model does not introduce any additional computational cost in deployment.

In the following parts, we discuss how to effectively optimize CySGAN with multiple objectives and data augmentations. Different from standard unsupervised image translation, the two domains are *asymmetric*, as X is labeled, while Y is unlabeled. We thus apply similar image translation losses but unique segmentation losses for X and Y domains.

### B. Image Translation Losses

Given an input image $x_{i}∼I_{X}$, we can denote F as the *forward* generator and G as the *backward* generator (Eqn. 1). Since paired $I_{X}$ and $I_{Y}$ are difficult or even infeasible to obtain, F is usually optimized using the *adversarial* loss so that the real and synthesized images gradually become indistinguishable in terms of distribution:

(2) $${ℒ}_{GAN}\left(F,D_{Y}^{I}\right)=\log D_{Y}^{I}\left(y_{i}\right)+\log\left(1-D_{Y}^{I}\left({\overset{ˆ}{y}}_{i}\right)\right)$$

where $D_{Y}^{I}$ is the $I_{Y}$ discriminator, while $y_{i}$ and ${\overset{ˆ}{y}}_{i}$ are true and synthesized images $\left({\overset{ˆ}{y}}_{i}=F{\left(x_{i}\right)}_{\left[I\right]}\right)$, respectively. Following CycleGAN [9], we additionally use a backward generator G and discriminator $D_{X}^{I}$ for $I_{X}$ to symmetrically optimize ${ℒ}_{GAN}\left(G,D_{X}^{I}\right)$ for translating $I_{Y}$ to $I_{X}$, as well as enforcing the *cycle-consistency* loss for the images in both domains:

(3) $${ℒ}_{\text{cyc}}\left(F,G\right)={∥G{\left({\overset{ˆ}{y}}_{i}\right)}_{\left[I\right]}-x_{i}∥}_{1}+{∥F{\left({\overset{ˆ}{x}}_{i}\right)}_{\left[I\right]}-y_{i}∥}_{1}$$

The GAN and cyclic losses enable the models to transfer images between $I_{X}$ and $I_{Y}$ distributions. However, the training of the original binary cross-entropy GAN loss (Eqn. 2) can be unstable. Therefore, following the official CycleGAN implementation, we instead optimize the LSGAN [28] loss:

(4) $${ℒ}_{LSGAN}\left(F,D_{Y}^{I}\right)={\left(D_{Y}^{I}\left(y_{i}\right)-1\right)}^{2}+{\left(D_{Y}^{I}\left({\overset{ˆ}{y}}_{i}\right)+1\right)}^{2}$$

This loss formulation has been shown to prevent vanishing gradient and smooth the training process. A symmetric adversarial loss is applied to optimize G. In our proposed CySGAN, the image translation losses do not affect the output layers for the segmentation maps, but it does change the backbone shared by both translation and segmentation modules.

### C. Instance Segmentation Losses

#### 1) Labeled Source Domain:

**Fig. 3.**
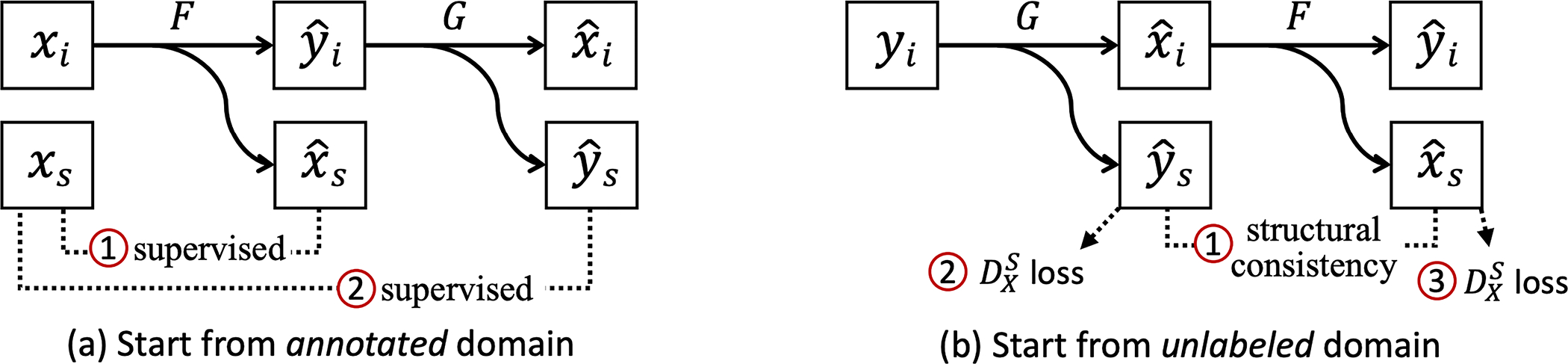
Different segmentation losses for two domains. (**a**) For an annotated image in ***X***, we compute the supervised losses of predicted segmentation representations against the label. (**b**) For an unlabeled image in ***Y***, we enforce *structural consistency* between predicted representations (as the underlying structures should be shared) and also segmentation-based adversarial losses to improve the quality of predictions in the absence of paired labels.

Instance segmentation approaches for microscopy images [3]–[5], [21] usually predict instance representations computed from the permutation-invariant labels and then apply a decoding algorithm to yield the masks. In this work, we follow U3D-BCD [5] that predicts the *binary foreground mask* (B), *instance contour map* (C), and *signed distance transform* (D) as three output channels using a 3D U-Net [7], which are decoded by a marker-controlled watershed (MW) algorithm. The B and C channels are optimized with the binary cross-entropy loss (BCE), while D is regressed with the mean squared error (MSE). Given an image-label pair $\left(x_{i},x_{s}\right)$ sampled from $\left(I_{X},S_{X}\right)$, the loss is

(5) $${ℒ}_{seg\;}\left(F\right)={ℒ}_{bce\;}\left(F{\left(x_{i}\right)}_{\left[\text{S}\right]}^{B},x_{s}^{B}\right)+{ℒ}_{bce}\left(F{\left(x_{i}\right)}_{\left[\text{S}\right]}^{C},x_{s}^{C}\right)\;\;+{\left\|F{\left(x_{i}\right)}_{\left[\text{S}\right]}^{D}-x_{s}^{D}\right\|}_{2}^{2}$$

where $x_{s}=\left[x_{s}^{B},x_{s}^{C},x_{s}^{D}\right]$ is the concatenation of the three representations. For the supervised direction, the segmentation loss ${ℒ}_{\text{seg}}(F)$ of the forward generator and segmentation loss ${ℒ}_{\text{seg}}(G)$ (based on the synthesized ${\overset{ˆ}{y}}_{i}$) of the backward generator are optimized by directly comparing ${\overset{ˆ}{x}}_{s}$ and ${\overset{ˆ}{y}}_{s}$ with $x_{s}$ from $S_{X}$ ① and ② in Fig. 3a).

The loss ${ℒ}_{\text{seg}}(G)$ effectively trains G in a supervised manner to predict the segmentation representations. Moreover, this design is not restricted to a particular set of instance representations and can be easily modified to incorporate other methods^4^. In the next part, we present a set of novel losses to better leverage the *unlabeled* domain Y.

#### 2) Unlabeled Target Domain:

Since Y is unlabeled, it is impossible to apply the supervised losses that we applied to X. To further improve segmentation quality, we introduce a *structural consistency* loss between the segmentation outputs of both generators, ${\overset{ˆ}{y}}_{s}$ and ${\overset{ˆ}{x}}_{s}$ ① Fig. 3b), as they should share identical underlying structures even if the inputs are from two modalities. This loss ${ℒ}_{sc}(F,B)$ is formulated as

(6) $${ℒ}_{sc}(F,G)={∥G{\left(y_{i}\right)}_{\left[S\right]}-F{\left(G{\left(y_{i}\right)}_{\left[I\right]}\right)}_{\left[S\right]}∥}_{1}$$

On the other hand, since we have unpaired instance segmentation masks $S_{X}$ of neuronal nuclei in a different modality, we also add structure-based adversarial losses to the predictions ② and ③ in Fig. 3b) to enforce their distributional similarity with $S_{X}$, which are denoted as ${ℒ}_{LSGAN}\left(G,D_{X}^{S}\right)$ and ${ℒ}_{LSGAN}\left(F,D_{X}^{S})\right.$ (see the LSGAN formulation in Eqn. 4). Please note that this loss requires similar dimensions for the instances in both datasets (*i.e*., the resolutions have to match), and we will elaborate our preprocessing steps in Sec. IV. Specifically, the discriminator $D_{X}^{S}$ takes the concatenation of all three representations to emphasize the correlation between them, as the representations are calculated from the same instance masks. This design also avoids using three independent discriminators that increase the system complexity. The architecture of $D_{X}^{S}$ is almost identical to the image discriminators except for the number of input channels. In summary, the structural consistency loss and segmentation-based adversarial losses provided additional supervision in the absence of paired labels for $I_{Y}$.

Our method is connected to *semi-supervised* learning as we incorporate unlabeled images in optimization using losses without paired labels. We can also choose other semi-supervised objectives, *e.g*., augmentation consistency [29], when the model takes images in the unlabeled domain as inputs. Our work emphasizes the concept of leveraging unlabeled images in a unified translation-segmentation framework, while the specific design choices can vary.

### D. Implementation

#### 1) Full Objective:

The full objective (ℒ) of CySGAN is the sum of losses in Sec. III-B and III-C, which is

(7) $$ℒ=\underset{\text{image-to-image}\;\text{translation}}{\underbrace{{ℒ}_{GAN}\left(F,D_{Y}^{I}\right)+{ℒ}_{GAN}\left(G,D_{X}^{I}\right)+{ℒ}_{cyc}(F,G)}}\;\;+\underset{\text{supervised}\;\text{segmentation}}{\underbrace{{ℒ}_{\text{seg}}(F)+{ℒ}_{\text{seg}}(G)}}\;\;+\underset{\text{semi-supervised}\;\text{segmentation}}{\underbrace{{ℒ}_{\text{sc}}(F,G)+{ℒ}_{GAN}\left(G,D_{X}^{S}\right)+{ℒ}_{GAN}\left(F,D_{X}^{S}\right)}}$$

We assign a uniform weight for all losses without tweaking. In the ablation studies, we also test a CySGAN model without the semi-supervised segmentation loss to demonstrate its effectiveness to the framework.

#### 2) Augmentation-Aware Cycle Consistency:

**Fig. 4.**
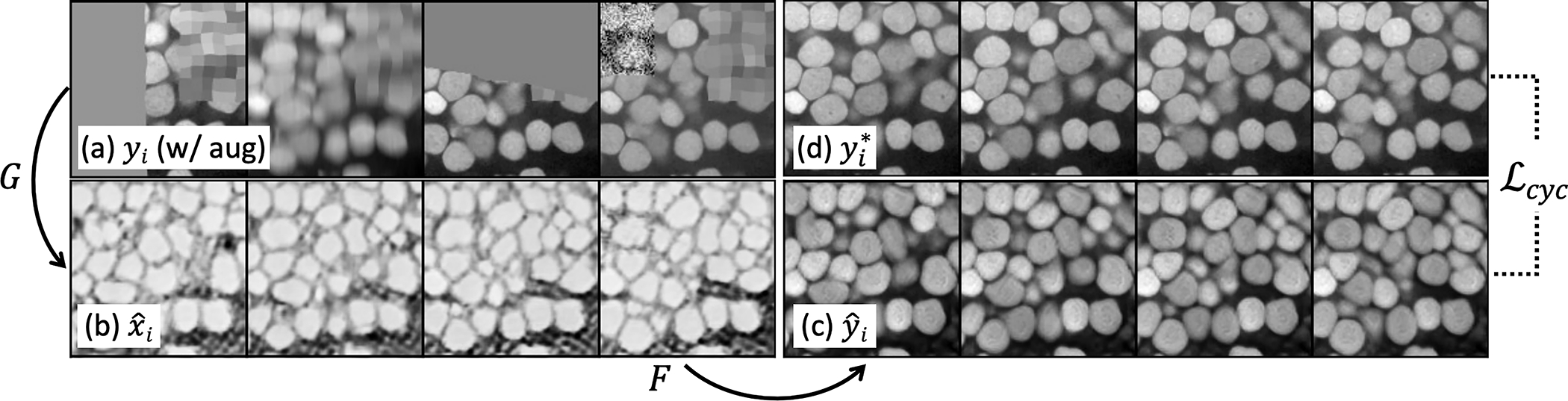
Restore augmented regions with an adapted cycle-consistency strategy. We show four consecutive slices of (**a**) augmented real $I_{Y}$ input, (**b**) synthesized $I_{X}$ volume, (**c**) reconstructed $I_{Y}$ volume and (**d**) real $I_{Y}$ volume w/o augmentations. By forcing the cycle consistency of (c) to (d), the model learns to restore corrupted regions with 3D context.

The U3D-BCD [5] model uses multiple training augmentations like random missing, blurry and noisy regions (Fig. 4a). We keep them in CySGAN for better segmentation quality. However, the image discriminator can easily distinguish synthesized images from real ones if the augmentations are clearly noticeable in the translated ones, breaking the balance in GAN training. Therefore, we propose an upgraded cycle consistency (Eqn. 3) by streaming the training images for X and Y in both augmented and clean (unaugmented) forms. As shown in Fig. 4 (each subfigure shows consecutive slices of a 3D volume), G transfers augmented $y_{i}$ to ${\overset{ˆ}{x}}_{i}$, and F reconstructs ${\overset{ˆ}{x}}_{i}$ to ${\overset{ˆ}{y}}_{i}$. Instead of calculating ${ℒ}_{cyc}(F,G)$ of ${\overset{ˆ}{y}}_{i}$ to $y_{i}$, we enforce its similarity to the clean $y_{i}^{*}$ (Fig. 4d). By using the augmentation-aware cycle consistency strategy, both generators learn to restore corrupted regions using 3D context^5^ in addition to image translation. We show in the ablation studies that this strategy has a significant impact on the domainadaptive segmentation performance.

#### 3) Network Details and Optimization:

We use 3D U-Nets [7] for F and G. They have identical architectures, but the parameters are not shared, which is similar to CycleGAN. Each network has one input channel and four output channels for the translated image and BCD segmentation representations (Fig. 2). For the GAN objectives, we use 3D convolutional discriminators, where the image discriminators $D_{X}^{I}$ and $D_{Y}^{I}$ have a single input channel for the gray-scale images, while the segmentation-based discriminators $D_{X}^{S}$ has three input channels for the BCD representations. Each discriminator has five layers, where each one consists of a strided convolution, a batch normalization, and a non-linear activation. Following PatchGAN [16], the final layer outputs a single-channel feature map representing the *realness* of corresponding input patches. The idea is to evaluate the generator’s performance at the level of local image patches rather than applying a coarse global penalty. As discussed in Sec. III-B, we optimize the LSGAN objective (Eqn. 4) instead of the BCE GAN loss (Eqn. 2) for training stability. When calculating the segmentation losses, we detach the synthesized image to avoid the segmentation objectives affecting the image translation results.

We train the CySGAN model for 10^6^ iterations using the AdamW [30] optimizer with an initial learning rate of 2×10^−3^ (decreased with cosine annealing) and batch size of 8 using 4 NVIDIA V100 GPUs. Our implementation of the proposed CySGAN framework is based on the *PyTorch Connectomics* [31] open-source framework.

## IV. Datasets

**Fig. 5.**
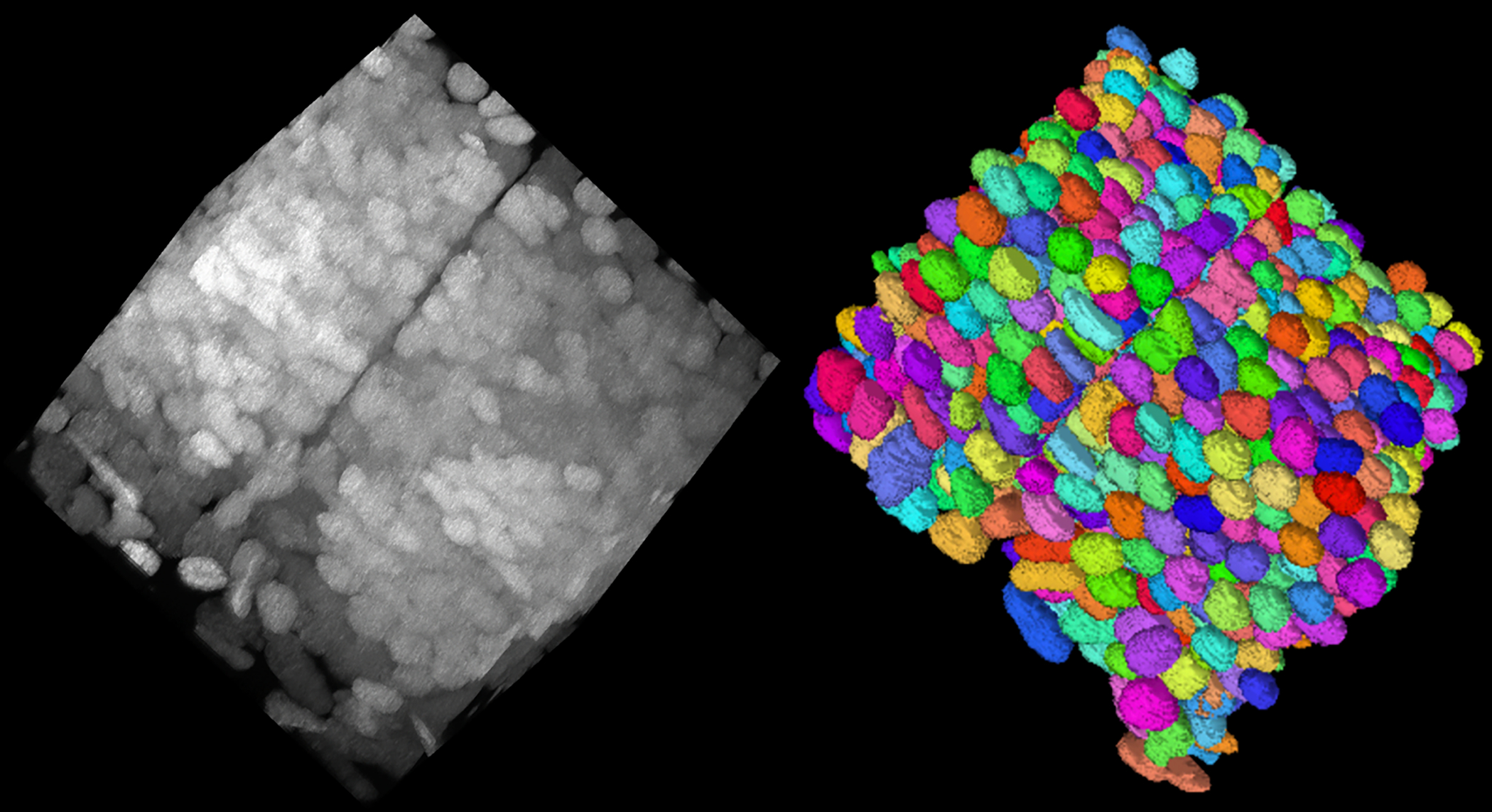
Visualization of the NucExM dataset. We sample a sub-volume of size (**1024, 1024, 100**) from the $V_{1}$ volume of NucExM. (Left) The expansion microscopy (ExM) image volume visualized using *Napari*. (Right) The corresponding 3D segmentation masks visualized using *Neuroglancer*.

As discussed in related works, existing domain-adaptive segmentation models are mainly developed for 2D segmentation and semantic segmentation. To alleviate the lack of benchmark datasets for 3D domain-adaptive instance segmentation in microscopy image analysis, we also release a fully annotated dataset with dense 3D neuronal nuclei instances (Fig. 5).

### 1) NucExM Dataset (Target):

We curated the saturated nuclei segmentation annotation for two expansion microscopy (ExM) [8] volumes by two neuroscience experts from a day 7 post-fertilization (dpf) zebrafish brain^6^, imaged with confocal microscopy. These volumes have an anisotropic resolution of 0.325×0.325×2.5 *μ*m in (x,y,z) order, with an approximate tissue expansion factor of 7.0. Thus the effective resolution becomes 0.046×0.046×0.357 *μ*m. The two volumes are of size 2048×2048×255 voxels with 9.6K and 8.8K nuclei, respectively (Table I). We downsample the volumes by ×4 along x and y axes to 512×512×255 to save computational cost during training and inference.

### 2) Source Dataset:

We use the NucMM-Z electron microscopy (EM) volume from the NucMM dataset [5] as the source data $\left(I_{X}\right.$ and $S_{X}$ in Fig. 1a). The original NucMM-Z covers nearly a whole zebrafish brain at a resolution of 0.48×0.48×0.48 *μ*m. Considering the different resolutions of the source and target datasets, we crop a 200 × 200 × 255 subvolume from NucMM-Z and upsample it to 512×512×255 to (roughly) match the resolution. The processed volume contains 12K neuronal nuclei instances. We also apply Gaussian filtering and thresholding of the instance masks after nearest-neighbor upsampling to smooth the boundaries.

### 3) Datasets Comparison:

**Fig. 6.**
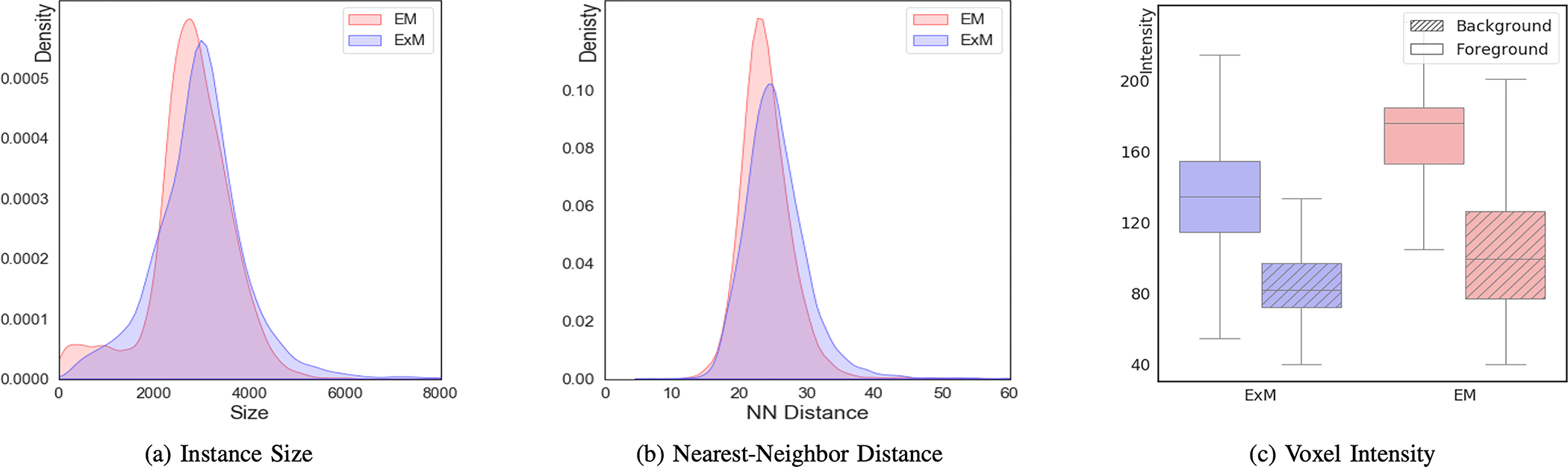
Statistics of the source (EM) and target (ExM) datasets. We show the distribution of (**a**) instance size (in terms of voxels) and (**b**) nearest-neighbor distance between nuclei centers. The density plots are normalized by the total number of instances in each volume. We also show (**c**) the voxel intensity distribution in object (foreground) and non-object (background) regions for both volumes. The domain gap is characterized by different intensity distributions and contrast.

Fig. 6 shows the comparison between the source (EM) and target (ExM) datasets. After downsampling of the target dataset and upsampling of the source dataset, the instance size (Fig. 6a) and nearest-neighbor distance between nuclei centers (Fig. 6b) roughly match, which is expected to help the model learn to segment 3D neuronal nuclei instances in a domain-adaptive setting. The domain gap is mainly characterized by the different intensity and contrast of object and non-object voxels (Fig. 6c). We show in experiments that the difference in appearance can hardly be solved by traditional appearance-level adaptation approaches like histogram matching.

### 4) Evaluation Metric:

Following common practice in instance segmentation [32], [33], we choose average precision (AP) as the evaluation metric. Specifically, for our 3D volumetric data, we choose AP-50 (*i.e*., AP with an IoU threshold of 0.5) and use the existing public implementation with improved efficiency for 3D volumes [21].

## V. Experiments

### A. Methods in Comparison

We compare CySGAN with three types of models targeting the segmentation of a new domain without any indomain annotation, including generalist models, appearance-level adaptation models, and feature-level adaptation models.

#### 1) Generalist models:

We compare with Cellpose [3] and StarDist [4] models using their official implementation. Cellpose predicts the flow-field representations for instances using neural networks, while StarDist predicts 3D star-convex polyhedra representations. Those models are pretrained on various training datasets covering different imaging modalities and species (*e.g*., the Cellpose model was pretrained on datasets with over 70k segmented objects). To improve the fairness in performance comparison, we conducted hyper-parameter tuning of the algorithms (*e.g*., the estimated diameters of the objects) to ensure the quality of the predictions.

#### 2) Appearance-level adaptation:

Appearance-level adaptation approaches are the models that first translate images to the target appearance for training a segmentation model. Since existing approaches are mainly developed for 2D semantic segmentation [10], [11], [13] but rarely explore 3D instance segmentation, we implemented two kinds of baseline models that conduct translation and 3D instance segmentation sequentially. Specifically, we test both histogram matching (a traditional method) and CycleGAN [9] (a deep learning-based method) as the translation module. We use U3D-BCD [5] for segmentation, which is consistent with the CySGAN generators but without the output channel for translated images. Moreover, we test the $I_{X}\to I_{Y}$ version that transfers $I_{X}$ to $I_{Y^{\prime }}$ and trains a model in the target domain using synthesized images, and $I_{Y}\to I_{X}$ that transfers $I_{Y}$ to $I_{X^{\prime }}$ and predicts the segmentation using a model trained in the source. Note that $I_{X}\to I_{Y}$ adaptation is usually preferred as the $I_{Y}\to I_{X}$ approach needs to run the image translation module as inference time, introducing additional computational cost.

#### 3) Feature-level adaptation:

Appearance-level adaptation models described before first translates images between the source and target domains. In comparison, feature-level domain adaptation models commonly map the source and target distributions in the model embedding space. For feature-level domain adaptation, we implemented a model sharing a similar high-level idea as Tsai *et al*. [27]. Specifically, based on the same U3D-BCD model in the appearance-level adaptation models and our CySGAN, we apply the first GAN loss to match the distribution of source and target predictions (*i.e.*, the BCD segmentation representations) and the second GAN loss to align the target features to the source features in the embedding space of the 3D U-Net model. Other training details, including data augmentations, are the same as the segmentation modules in the appearance-level adaptation models.

### B. Results

Since there are two volumes in the NucExM dataset, we only use one volume $\left(V_{1}\right)$ to optimize the model while running inference on $V_{1}$ and $V_{2}$. The inference results of $V_{2}$, therefore, demonstrate the model’s generalization ability. Note that since the setting is unsupervised domain-adaptation, only the ExM images of $V_{1}$ are used in training without any annotations. Table II summarizes the results. Our CySGAN outperforms pretrained generalist models, feature-level adaptation models, and appearance-level adaptation models with either histogram matching or CycleGAN for image translation. Specifically, CySGAN outperforms the second-best model (CycleGAN+Segm, $I_{X}\to I_{Y}$) by absolutely 5.7%, demonstrating the effectiveness of our proposed framework. The results also show that $I_{X}\to I_{Y}$ versions generally perform better than $I_{Y}\to I_{X}$ ones in sequential models. Please note that, although the models are not optimized on $V_{2}$, all methods generally perform better on $V_{2}$ as the volume is relatively easier to segment.

**Fig. 7.**
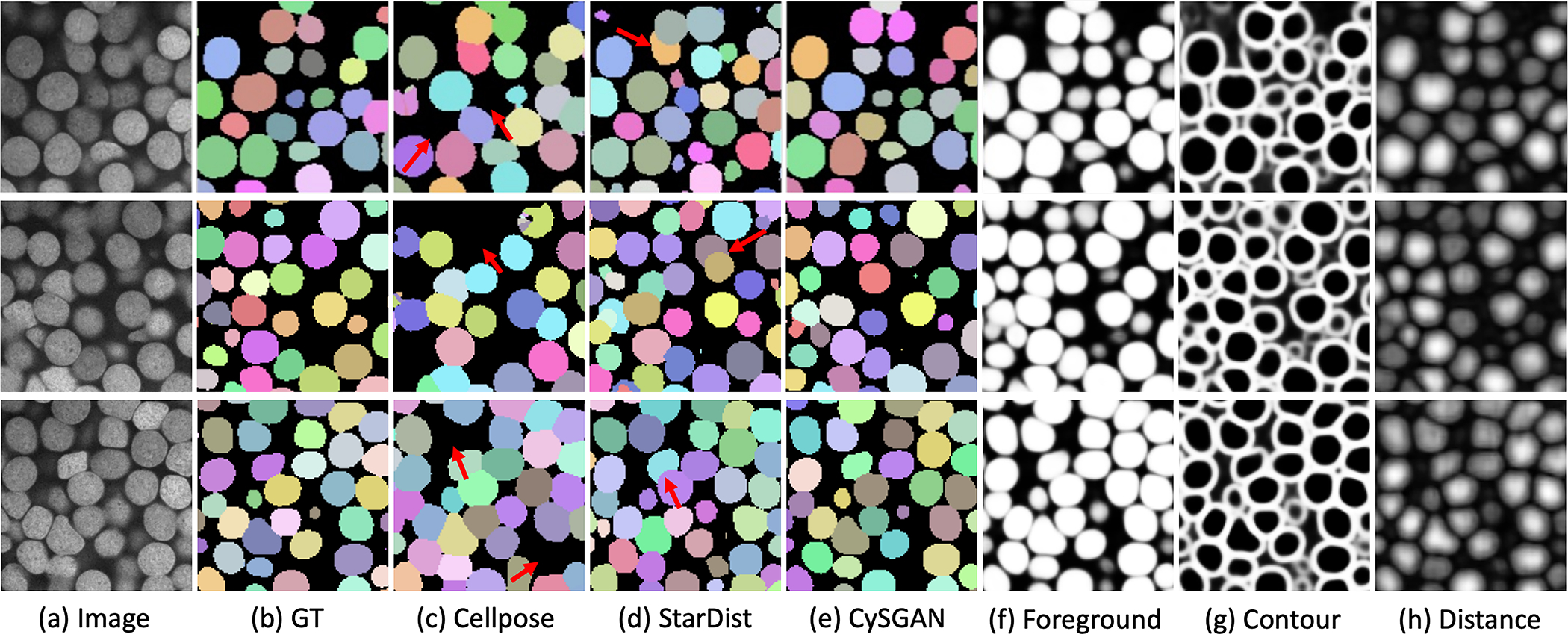
Visual comparisons of segmentation results. (**a**) ExM image, (**b**) ground-truth instances, (**c**) Cellpose [3], (**d**) StarDist [4] and (**e**) CySGAN results. The red arrows highlight false negatives in Cellpose predictions and overlapping masks from StarDist. We also show (**f-h**) the predicted segmentation representations of U3D-BCD used in CySGAN. Note that all the nuclei instances are 3D as shown in Fig. 5. We present representative 2D slices in this visualization to demonstrate the model performance.

The visual results in Fig. 7 show that Cellpose’s segmentation has obvious false negatives, as highlighted by the red arrows. From our hyperparameter search for Cellpose, we found that the challenging contrast of the ExM data causes missing foreground predictions. StarDist’s masks, on the other hand, tend not to align well with instance boundaries and overlap with each other, which are also highlighted using red arrows. We empirically find that the strong star-convex shape prior often overlooks other features like boundaries and thus struggles with non-spherical shapes. Our CySGAN model that combines three predicted mask representations (Fig. 7, f–h) yields favorable 3D instance segmentation results.

### C. Ablation Studies

We further validate three important design choices of CySGAN, including the data augmentations (Fig. 4), semi-supervised segmentation losses for the *unlabeled* domain (Eq. 7), and learning the BCD [5] representation.

Table III shows the results when removing those components from the CySGAN model on the $V_{1}$ NucExM image volume. First, without data augmentations and the corresponding cycle-consistency loss to restore corrupted regions, the performance is significantly degraded by 16.6%. We also observe that the model is prone to model collapse (*i.e*., the generator tends to generate a single pattern during the optimization) without data augmentations. Therefore our training strategy can improve both the performance and robustness of the domain-adaptive segmentation model. Second, CySGAN without the semi-supervised segmentation losses (which can be regarded as a 3D instance segmentation version of SUSAN [14]), the performance is decreased by 4.9% and similar to the result of the model sequentially conducting image translation and segmentation (CycleGAN + Segm in Table II). Third, we also test a model that only learns the binary foreground mask and contour map (BC), as in Wei *et al*. [21], without the signed distance map in the BCD representation [5]. The discriminator for the segmentation-based GAN loss is updated accordingly to have two input channels without modifying other training protocols. The BC version is worse than the default CySGAN model by 8.4%, validating the importance of the signed distance map in segmenting closely-touching 3D instances. Those results demonstrate the essentiality of those components in CySGAN and also provide informative data points to quantify the importance of those designs.

## VI. Conclusion

In this work, we present CySGAN, a unified domainadaptive segmentation framework optimized with image translation losses as well as supervised and semi-supervised instance segmentation losses to tackle an unlabeled imaging modality. CySGAN outperforms and simplifies models that conduct translation and segmentation using separate networks. We also publicly release the NucExM dataset as a testbed for future domain-adaptive 3D instance segmentation models. In our application scenario, the morphology of the source and target objects are relatively close. Thus, important future directions include segmenting modalities where the instance structures differ significantly from those in the source domain.

## Figures and Tables

**TABLE I:** NucExM Dataset metadata. We curated and densely annotated a *neuronal nuclei* segmentation dataset with two ExM volumes of zebrafish. The tissue was expanded by about **7**× to increase resolution.

Sample	#Volumes	Volume Size (each)	Resolution (μm)	Ex. Ratio	#Instances

Zebrafish Brain	2	2048×2048×255	0.325×0.325×2.5	7.0	9.6K+8.8K

**TABLE II:** Benchmark results on the NucExM dataset. We compare CySGAN with pretrained generalist models, feature-level adaptation models, and appearance-level adaptation models using the AP-50 scores. except for the generalist models, all other approaches use u3d-bcd [5] for segmentation. **Bold** and underlined numbers denote the 1st and 2nd results.

Method	Cellpose	StarDist	Feat. DA	Histogram + Segm		CycleGAN + Segm		CySGAN (Ours)
				*I_X_* → *I_Y_*	*I_Y_* → *I_X_*	*I_X_* → *I_Y_*	*I_Y_* → *I_X_*	

AP-50 (*V*_1_)	0.644	0.816	0.774	0.807	0.804	0.867	0.772	**0.927**
AP-50 (*V*_2_)	0.765	0.875	0.795	0.826	0.816	0.881	0.777	**0.934**

Average	0.705	0.846	0.785	0.817	0.810	0.874	0.775	**0.931**

**TABLE III:** Ablation studies of CySGAN. The results show obvious performance degradation without using data augmentations, semi-supervised losses, and signed distance map (D), demonstrating the importance of those components for CySGAN.

Configuration	w/o Augmentation	w/o Semi-sup Losses	w/ BC only	CySGAN (Ours)

AP-50 (*V*_1_)	0.761 (−0.166)	0.878 (−0.049)	0.843 (−0.084)	**0.927**

## Footnotes

1 Expansion microscopy [8] alleviates the resolution limitation in optical microscopy by physically expanding the tissues.

2 The opposite way, which transfers the target domain images to the source domain and applies a supervised model trained on the source data, is also reasonable. However, the community uses it less often as this direction requires both the translation and segmentation models at inference time.

3 The source-to-target generator is optimized jointly during training but not needed at inference time.

4 For example, SUSAN [14] applies the supervised segmentation losses for 2D semantic masks with pixel-wise class annotations.

5 The strong missing-region augmentation is not applied to successive sections to facilitate using 3D context in translation and segmentation.

6 All procedures involving animals at the Massachusetts Institute of Technology (MIT) were conducted in accordance with the US National Institutes of Health Guide for the Care and Use of Laboratory Animals and approved by the MIT Committee on Animal Care. The IACUC protocol number is 1221–100-24, which was approved on 12/23/2021.

## References

[1] Rivron NC, Frias-Aldeguer J, Vrij EJ, Boisset J-C, Korving J, Vivié J, Truckenmüller RK, Van Oudenaarden A, Van Blitter-swijk CA, and Geijsen N, “Blastocyst-like structures generated solely from stem cells,” Nature, vol. 557, no. 7703, pp. 106–111, 2018. 29720634 10.1038/s41586-018-0051-0

[2] Caicedo JC, Goodman A, Karhohs KW, Cimini BA, Ackerman J, Haghighi M, Heng C, Becker T, Doan M, McQuin C , “Nucleus segmentation across imaging experiments: the 2018 data science bowl,” Nature methods, vol. 16, no. 12, pp. 1247–1253, 2019. 31636459 10.1038/s41592-019-0612-7 PMC6919559

[3] Stringer C, Wang T, Michaelos M, and Pachitariu M, “Cellpose: a generalist algorithm for cellular segmentation,” Nature Methods, vol. 18, no. 1, pp. 100–106, 2021. 33318659 10.1038/s41592-020-01018-x

[4] Weigert M, Schmidt U, Haase R, Sugawara K, and Myers G, “Star-convex polyhedra for 3d object detection and segmentation in microscopy,” in Proceedings of the IEEE/CVF Winter Conference on Applications of Computer Vision, 2020, pp. 3666–3673.

[5] Lin Z, Wei D, Petkova MD, Wu Y, Ahmed Z, Zou S, Wendt N, Boulanger-Weill J, Wang X, Dhanyasi N , “Nucmm dataset: 3d neuronal nuclei instance segmentation at sub-cubic millimeter scale,” in International Conference on Medical Image Computing and Computer-Assisted Intervention. Springer, 2021, pp. 164–174.

[6] Ronneberger O, Fischer P, and Brox T, “U-net: Convolutional networks for biomedical image segmentation,” in MICCAI. Springer, 2015, pp. 234–241.

[7] Çiçek Ö, Abdulkadir A, Lienkamp SS, Brox T, and Ronneberger O, “3d u-net: learning dense volumetric segmentation from sparse annotation,” in MICCAI. Springer, 2016, pp. 424–432.

[8] Chen F, Tillberg PW, and Boyden ES, “Expansion microscopy,” Science, vol. 347, no. 6221, pp. 543–548, 2015. 25592419 10.1126/science.1260088 PMC4312537

[9] Zhu J-Y, Park T, Isola P, and Efros AA, “Unpaired image-to-image translation using cycle-consistent adversarial networks,” in Proceedings of the IEEE international conference on computer vision, 2017, pp. 2223–2232.

[10] Hoffman J, Tzeng E, Park T, Zhu J-Y, Isola P, Saenko K, Efros A, and Darrell T, “Cycada: Cycle-consistent adversarial domain adaptation,” in International conference on machine learning. PMLR, 2018, pp. 1989–1998.

[11] Huo Y, Xu Z, Bao S, Assad A, Abramson RG, and Landman BA, “Adversarial synthesis learning enables segmentation without target modality ground truth,” in 2018 IEEE 15th international symposium on biomedical imaging (ISBI 2018). IEEE, 2018, pp. 1217–1220. 10.1109/isbi.2018.8363790 PMC12462902 41018946

[12] Januszewski M and Jain V, “Segmentation-enhanced cyclegan,” bioRxiv, p. 548081, 2019.

[13] Chen C, Dou Q, Chen H, Qin J, and Heng PA, “Unsupervised bidirectional cross-modality adaptation via deeply synergistic image and feature alignment for medical image segmentation,” IEEE transactions on medical imaging, vol. 39, no. 7, pp. 2494–2505, 2020. 32054572 10.1109/TMI.2020.2972701

[14] Liu F, “Susan: segment unannotated image structure using adversarial network,” Magnetic resonance in medicine, vol. 81, no. 5, pp. 3330–3345, 2019. 30536427 10.1002/mrm.27627 PMC7140982

[15] Lee K, Zung J, Li P, Jain V, and Seung HS, “Superhuman accuracy on the snemi3d connectomics challenge,” arXiv:1706.00120, 2017.

[16] Isola P, Zhu J-Y, Zhou T, and Efros AA, “Image-to-image translation with conditional adversarial networks,” in Proceedings of the IEEE conference on computer vision and pattern recognition, 2017, pp. 1125–1134.

[17] Goodfellow I, Pouget-Abadie J, Mirza M, Xu B, Warde-Farley D, Ozair S, Courville A, and Bengio Y, “Generative adversarial nets,” Advances in neural information processing systems, vol. 27, 2014.

[18] Liu M-Y, Breuel T, and Kautz J, “Unsupervised image-to-image translation networks,” Advances in neural information processing systems, vol. 30, 2017.

[19] Pang Y, Lin J, Qin T, and Chen Z, “Image-to-image translation: Methods and applications,” IEEE Transactions on Multimedia, 2021.

[20] Ciresan D, Giusti A, Gambardella LM, and Schmidhuber J, “Deep neural networks segment neuronal membranes in electron microscopy images,” in NeurIPS, 2012, pp. 2843–2851.

[21] Wei D, Lin Z, Franco-Barranco D, Wendt N , “Mitoem dataset: Large-scale 3d mitochondria instance segmentation from em images,” in International Conference on Medical Image Computing and Computer-Assisted Intervention. Springer, 2020, pp. 66–76. 10.1007/978-3-030-59722-1_7 PMC7713709 33283212

[22] Turaga SC, Briggman KL, Helmstaedter M, Denk W, and Seung HS, “Maximin affinity learning of image segmentation,” in NeurIPS, 2009, pp. 1865–1873.

[23] Cousty J, Bertrand G, Najman L, and Couprie M, “Watershed cuts: Minimum spanning forests and the drop of water principle,” TPAMI, vol. 31, pp. 1362–1374, 2008. 10.1109/TPAMI.2008.173 19542572

[24] Zlateski A and Seung HS, “Image segmentation by size-dependent single linkage clustering of a watershed basin graph,” arXiv:1505.00249, 2015.

[25] Krasowski N, Beier T, Knott G, Köthe U, Hamprecht FA, and Kreshuk A, “Neuron segmentation with high-level biological priors,” TMI, vol. 37, no. 4, 2017. 10.1109/TMI.2017.2712360 28600240

[26] Chartsias A, Joyce T, Dharmakumar R, and Tsaftaris SA, “Adversarial image synthesis for unpaired multi-modal cardiac data,” in International workshop on simulation and synthesis in medical imaging. Springer, 2017, pp. 3–13.

[27] Tsai Y-H, Hung W-C, Schulter S, Sohn K, Yang M-H, and Chandraker M, “Learning to adapt structured output space for semantic segmentation,” in Proceedings of the IEEE conference on computer vision and pattern recognition, 2018, pp. 7472–7481.

[28] Mao X, Li Q, Xie H, Lau RY, Wang Z, and Paul Smolley S, “Least squares generative adversarial networks,” in Proceedings of the IEEE international conference on computer vision, 2017, pp. 2794–2802.

[29] Sohn K, Berthelot D, Carlini N, Zhang Z, Zhang H, Raffel CA, Cubuk ED, Kurakin A, and Li C-L, “Fixmatch: Simplifying semi-supervised learning with consistency and confidence,” Advances in Neural Information Processing Systems, vol. 33, pp. 596–608, 2020.

[30] Loshchilov I and Hutter F, “Decoupled weight decay regularization,” arXiv preprint arXiv:1711.05101, 2017.

[31] Lin Z, Wei D, Lichtman J, and Pfister H, “Pytorch connectomics: A scalable and flexible segmentation framework for em connectomics,” arXiv preprint arXiv:2112.05754, 2021.

[32] Cordts M, Omran M, Ramos S, Rehfeld T, Enzweiler M, Benenson R, Franke U, Roth S, and Schiele B, “The cityscapes dataset for semantic urban scene understanding,” in Proceedings of the IEEE conference on computer vision and pattern recognition, 2016, pp. 3213–3223.

[33] Lin T-Y, Maire M, Belongie S, Hays J, Perona P, Ramanan D, Dollár P, and Zitnick CL, “Microsoft coco: Common objects in context,” in European conference on computer vision. Springer, 2014, pp. 740–755.

